# Pancreatitis as a Main Consequence of *APOC2*-Related Hypertriglyceridemia: The Role of Nonsense and Frameshift Variants

**DOI:** 10.1155/2024/6653857

**Published:** 2024-05-30

**Authors:** Bahareh Rabbani, Mohadeseh Aghli Moghadam, Shiva Esmaeili, Amirhassan Rabbani, Bahman Akbari, Nejat Mahdieh

**Affiliations:** ^1^ Growth and Development Research Center Tehran University of Medical Sciences, Tehran, Iran; ^2^ Department of Genetics Faculty of Sciences Shahid Chamran University of Ahvaz, Ahvaz, Iran; ^3^ Cardiogenetic Research Center Rajaie Cardiovascular Medical and Research Center Iran University of Medical Sciences, Tehran, Iran; ^4^ Taleghani Hospital Department of Transplant & Hepatobiliary Surgery Shahid Beheshti University of Medical Sciences, Tehran, Iran; ^5^ Department of Medical Biotechnology School of Medicine Kermanshah University of Medical Sciences, Kermanshah, Iran; ^6^ Physiology Research Center Iran University of Medical Sciences, Tehran, Iran

## Abstract

*APOC2-*related hypertriglyceridemia occurs due to biallelic variants of this gene. Here, genotype-phenotype architecture of all pathogenic *APOC2* variants is investigated among heterozygous and homozygous individuals. Clinical heterogeneity of various types of the variants is also described, and pancreatitis in more than half of homozygotes carrying chain-termination variants is highlighted as well. For this study, patients were selected who had a plasma triglyceride level above 250 mg/dL. The coding and intronic regions of the *APOC2* gene were amplified using the Sanger sequencing to investigate the presence of variants. The genotypes, lipid profiles, and detailed clinical features were documented for all *APOC2*-related patients and heterozygous individuals. Pathogenicity of the variants was predicted and categorized using available bioinformatics tools such as MutationTaster and PolyPhen-2 and ACMG criteria. MetaDome and Phyre2 were applied for structural and functional in silico analyses. 40% (12 out of 30) of *APOC2* variants were chain-termination (nonsense and frameshift) variants. These types of variants were determined in 60.53% of patients. 55% of these patients showed pancreatitis followed by lipemia retinalis (29%), abdominal pain (24%), hepatosplenomegaly (24%), and xanthomas (18%). The mean age of onset was about 22 years old. In at least 50% of 38 homozygous individuals, the TG level was more than 2000 mg/dL. More than 25% of heterozygous individuals showed at least one symptom. Pancreatitis and a severe form of HTG were found in 5 and 2% of heterozygous individuals, respectively. The main clinical features of *APOC2*-related hypertriglyceridemia include pancreatitis, lipemia retinalis, abdominal pain, hepatosplenomegaly, and xanthomas. Nonsense and frameshift homozygous variants usually lead to a severe form of hypertriglyceridemia. Pancreatitis is one of the main consequences of these types of mutations; thus, it is important to consider this point when evaluating asymptomatic individuals. Heterozygous individuals may become symptomatic due to the role of unknown modifying agent including environmental genetic factors.

## 1. Introduction

Dyslipidemia as a common clinical condition is an important risk factor predisposing cardiovascular disease with an estimated prevalence of 1 in 600, in the general population [1]. Clinical features of dyslipidemic patients may include lipemia retinalis, pancreatitis, eruptive xanthomas, milky plasma, nausea, vomiting, hepatosplenomegaly, and vague abdominal pain [2]. Less common symptoms include diarrhea and neurological problems such as seizures [3]. This condition can be due to genetic and/or nongenetic agents. Approximately 2% of these patients is due to different variants in *APOC2* gene [2]. Nongenetic factors include lifestyle such as consumption of high-fat foods and alcohol consumption and other factors such as obesity.

Hypertriglyceridemia (HTG) is defined as triglyceride (TG) levels above 175 mg/dL (2.0 mmol/L). There is some controversy regarding HTG threshold values and classification. Various committees have recommended relatively different threshold values and classified HTG as mild, “moderate,” and “severe” forms. According to Endocrine Society, we here define HTG as normal (>150), mild (150-199), moderate (200-999), severe (1000-1999), and very severe (<2000) form of HTG [4]. HTG is a major contributor of the risk of pancreatitis and atherosclerotic cardiovascular disease. It can increase the risk for high blood pressure, metabolic syndrome, and diabetes [5]. Familial chylomicronemia syndrome leads to severely elevated TG levels. Although the majority of adult HTG patients is due to a polygenic agents, severe HTG at a young age has monogenic causes [6]. It has been shown that it may occur due to additional effects of multiple variants in more than 40 genes [5, 7]. Familial hypercholesterolemia occurs due to pathogenic variant in *LDLR*, *APOB*, *PCSK9*, and *LDLRAP1*. Monogenic forms of familial HTG occur due to loss of function variants in lipoprotein lipase (*LPL*), apolipoprotein C2 (*APOC2*), apolipoprotein A5 (*APOA5*), *GPIHBPI*, and *LMF1* genes [8]. LPL, an enzyme encoded by the LPL gene, helps break down triglycerides carried by two different types of lipoproteins that transport fat from various organs to the bloodstream [6]. *APOA5* encodes apolipoprotein A-V (apoA-V) which is primarily associated with HDL, VLDL, and chylomicron particles. It is not associated with LDL in the blood [9]. *GPIHBPI* encodes glycosylphosphatidylinositol-anchored high-density lipoprotein-binding protein 1 which binds LPL and transports it to the capillary lumen [10]. *LMF1* encodes lipase maturation factor 1 protein required for the proper folding and maturation LPL protein [11].

*APOC2* gene encodes an essential coactivating factor for lipoprotein lipase digesting triglycerides [5]. This protein may have a more important role in HTG. Briefly, ApoC2, as an important component of chylomicrons, VLDLs, and HDLs, is essential for LPL activity. Without ApoC2, LPL activity is very low; however, the presence of this protein significantly increases the activity of LPL [6]. This protein has two domains: lipid-binding region located in the N-terminal and LPL-binding site located in the C-terminal helix. Thus, various variants of this gene may have different impacts. APOC2-related HTG is due to biallelic variants. Roughly 10% of individuals with severe HTG are heterozygous for loss of function variants in either *LPL* or *APOC2* genes [12]. In symptomatic heterozygous individuals, there may be a variety of symptoms caused by modifying agents that affect these particular genes.

Mutations in *APOC2* gene have been reported in several populations worldwide. Due to the lack of studies conducted in Iran, we decided to assess the presence of variations in this gene in the Iranian population as well. Previous studies have shown a high frequency of variations in the *APOC2* gene among individuals with dyslipidemia in the United States, Europe, and Eastern Asia, but no such data has been available for Iran. Thus, our study constitutes vital knowledge on the potential role of *APOC2* variations in dyslipidemia. Here, genotype-phenotype correlation in the *APOC2*-related HTG patients is highlighted and discussed. The genetic architecture of the disease among homozygous and heterozygous cases was determined. In addition, the results of screening of this gene are presented in an Iranian cohort.

## 2. Materials and Methods

### 2.1. Clinical Features and Biochemical Measurements of the Patients

All patients with *APOC2*-related HTG were recruited from the literature using a search conducted on available databases including HGMD and PubMed using the given terms (please see below). Genotype and clinical features of patients were recorded. In addition, data of heterozygotes were also analyzed. In silico analyses were applied to predict the pathogenicity of APOC2 variants. Genetic and clinical information of a cohort of Iranian patients were also investigated.

The number of homozygous and heterozygous individuals was extracted from the published studies to analyze the clinical characteristics including age, sex, hepatosplenomegaly, lipemia retinalis, eruptive xanthomas, pancreatitis, and abdominal pain. Profiles of TG and TC patents were also investigated. *APOC2* variants and the genotypes of patients were determined as well. Frequencies of different variants were checked in the studied populations. The subgroup analysis was done for different groups of HTG patients as well as domain-specific analysis of the variants.

### 2.2. Molecular Investigations

Fifty HTG patients referring to Rajaie Cardiovascular Center, Tehran, Iran, were also studied. Inclusion criteria were TG above 250 mg/dL. These patients were selected based on serum TG levels according to the blood test that was taken. The study was approved by the Ethics Committee of Iran University of Medical Sciences.

After taking an informed consent form, genomic DNA was extracted from peripheral blood leucocytes by a standard procedure. To assess quantity of DNA, a spectrophotometer (NanoDrop ND2000c, Thermo Scientific) was applied. All exons and introns, including the intron/exon boundaries of APOC2, were amplified using the forward and reverse primers (available upon request). Briefly, polymerase chain reaction (PCR) was executed in a final volume of 50 *μ*L, consisting of 10 pmol forward and reverse primers, 150 ng template DNA, 0.2 units/*μ*L Taq DNA polymerase, 1.5 mmol/L MgCl2, and 0.4 mmol/L of each dNTP in the reaction. The following PCR program was implemented: 5 minutes at 94°C initial denaturation and 30 cycles of 30 seconds at 94°C denaturation, 30 seconds at 63°C, and 30 seconds at 72°C extension, followed by 10 minutes at 72°C final extension. The PCR products were directly sequenced on an ABI PRISM™ 3500 (PE Applied Biosystems) sequencing analyzer using a BigDye termination method, and the reactions were analyzed on the GeneMapper software package.

### 2.3. Literature Review of Published *APOC2* Mutation Cases

A detailed comprehensive search was done in PubMed, Springer, John Wiley, and Elsevier inclusive until Jan 2023 using the keywords *APOC2*[ti/ab] variant AND/OR Apolipoprotein C-II[title/abstract] to find all the reported patients with *APOC2* pathogenic variants.

Demographic and clinical presentations, lipid profile (TG, TC), family history, phenotypic manifestations (HSM: hepatosplenomegaly, AS: asymptomatic, LR: lipemia retinalis, EX: xanthomas, Pan: pancreatitis, and AP: abdominal pain), and genotype/zygosity of individuals were extracted. Homozygous and heterozygous individuals were more investigated for being symptomatic and HTG forms. The variants were named using standard nomenclature and categorized into premature chain termination (PCT) including nonsense and frameshift variants, missense, gross deletions, and intronic and regulatory variants based on location and functional effect of the variants.

### 2.4. Bioinformatic Analyses

All *APOC2* likely/pathogenic variants according to the ClinVar were analyzed using available software tools to predict their pathogenicity. We used MutationTaster (http://www.mutationtaster.org/), CADD (https://cadd.gs.washington.edu/home), FATHMM (http://fathmm.biocompute.org.uk/), and PolyPhen-2 (http://genetics.bwh.harvard.edu/pph2/). The variants were classified using VarSome (https://varsome.com/). According to the guidelines provided by the American College of Medical Genetics and Genomics, sequence variants are categorized into interpretative categories based on expert opinion and empirical data. The interpretive categories are then used along with a systematic algorithm to determine the significance of the sequence variants as benign, likely benign, uncertain significance (VUS), likely pathogenic, and pathogenic [13].

A multiple amino acid sequence alignment of APOCII was performed using UniProt protein family members (UniProtKB/Swiss-Prot P02655). Pathogenicity analysis of *APOC2* variants was also done through aggregation of homologous human protein domains using MetaDome (https://stuart.radboudumc.nl/metadome/). MetaDome is a tool that makes use of the data from gnomAD and ClinVar to analyze mutation tolerance at each position in a human protein. It increases the analysis of your chosen gene by doing a parallel analysis of all homologous domains across the entire human genome. This helps to enhance the interpretation of the gene in the context of its full domain [14].

## 3. Result

### 3.1. Patient Demographics

#### 3.1.1. Iranian Cases

None of the Iranian patients showed pathogenic variants in *APOC2* gene. The study included 11 women and 39 men in the age range of 4 to 80 years (age average: 46 years), and patients' TG levels ranged from 250 to 776 mg/dL. Thirty patients were from Fars, six patients were from Azerbaijan, four patients were from Lorestan, four patients were from Kurdistan, three patients were from Mazandaran, one patient was from Gilan, and one patient was from Khuzestan.

Here, the data of *APOC2*-related HTG patents are presented according to their genotypes of *APOC2*. c.216-81T>C and c.216-145G>C were found each in 36% of patients.

Thirty-eight patients from twenty-seven families were found to have biallelic variants of *APOC2* gene, and thirty-nine individuals from 20 families were found to be heterozygotes (Tables 1 and 2). The mean age of the homozygous patients was 22.09 years. More than sixty-five percent was female. The majority of studied families were from European (14 of 27 families) and Asian (7 of 27 families) countries. The numbers of consanguineous and nonconsanguineous marriages were noted in 12 and 6 families, respectively.

#### 3.1.2. Symptomatic Heterozygous Individuals

**Figure 1:**
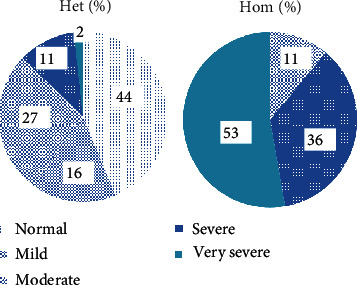
The frequencies of various groups of HTG homozygous and heterozygous individuals.

Ten of 39 heterozygous individuals were symptomatic. The mean TG and TC were 434.2 and 227.85 mg/dL, respectively (Table 1). The distribution of HTG frequencies across different groups is depicted in Figure 1. Approximately 44% of the individuals studied had TG levels that were within normal range followed by 27% of moderate HTG. However, severe HTG was observed in 2% of these subjects. Pancreatitis was also detected in 2 of the cases.

#### 3.1.3. Homozygous Patients

According to the Endocrine Society's diagnostic criteria for HTG, patients are categorized into mild, moderate, severe, very severe, and no data available groups. In our study, no individual was found to be in the mild HTG, moderate category was observed in 4 patients, 13 individuals fell within the severe HTG, and very severe category was detected in 18 cases. Also, TG level data was not available for 3 patients. More than fifty percent of these patients had TG more than 2000 mg/dL (very severe HTG). Other patients had severe (36%) and moderate (11%) HTG profile (Figure 1). The mean TG and TC were 4063.23 and 339.77 mg/dL, respectively. The distribution of clinical characteristics and their associated frequencies in the study subjects is illustrated in Figure 1. Seven subjects were asymptomatic in our study. Pancreatitis was the most prevalent symptom observed in these patients, occurring in 12 individuals, 7 of whom had premature termination variants, 4 had missense, and one patient's condition was caused by a regulatory variant. Lipemia retinalis was seen in 11 patients, 7 with premature termination variants, 2 with missense, and 2 with splice variants. AP was found in 9 patients, 6 of whom had premature termination variants, while 3 cases had missense mutations. HSM was identified in 9 cases, with 8 showing premature termination variants and 1 individual having a splice variant. EX was found in 7 individuals, 4 of whom had termination variants, 1 individual had a missense mutation, and regulatory and splicing variants were found in 1 patient each. HTG was the sole finding in 10 individuals.

### 3.2. Clinical Features in Different Types of Variants

A total of 23 patients had PCT variants, with mean TG and TC values of 4506.98 and 395.45, respectively. Of 9 HSM cases, 8 had PCT variants. In addition, PCT variants were detected in 7 out of 12 cases of pancreatitis and 7 out of 11 cases of lipemia retinalis.

**Figure 2:**
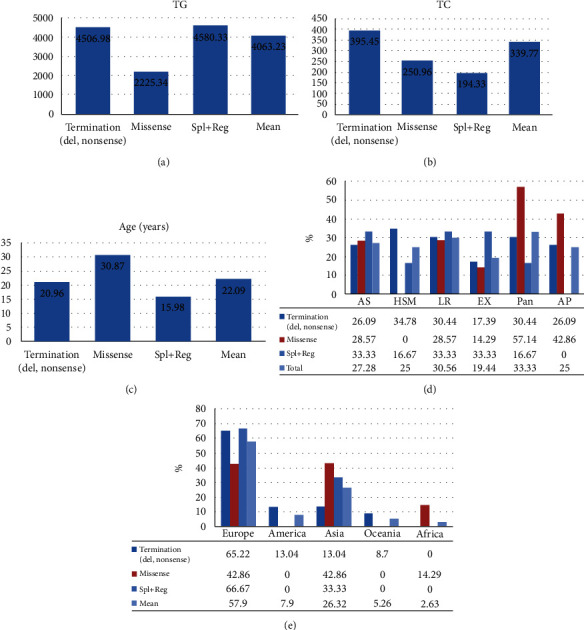
(a–c) TG (mg/dL), TC (mg/dL), and ages (years) of patients based on types of *APOC2* variants. The mean values of the patients are also shown. (d) Clinical features of patients with different types of variants. HSM: hepatosplenomegaly; AS: asymptomatic; LR: lipemia retinalis; EX: xanthomas: Pan: pancreatitis; AP: abdominal pain. (e) Distribution of variants among different continents.

The mean TG and TC levels among patients with missense variants were 2225.34 and 250.96, respectively. Missense variants were found in 4 patients with pancreatitis and 3 patients with AP. Meanwhile, the mean TG and TC levels for splice and regulatory variants were 4580.33 and 194.33, respectively. The mean age of these patients was 15.98 years as depicted in Figure 2.

A total of 30 variants in the APOC2 gene were found, comprising 8 missense, 6 nonsense, 6 indel, 4 splice, and 2 regulatory variants, along with 3 gross deletions and 1 gross duplication. In total, 12 variants were categorized as premature chain terminations (including nonsense and indel mutations) (Table 3). A detailed representation of the positions of the variants in different regions of the protein is displayed in Table 4 and Figure 2. Some protein regions had specific variant; O-glycosylated had nonsenses and lipid-binding region showed only missenses. The c.1A>G (p.Met1Val) and c.274C>T (p.Gln92Ter) were the most common variants, each found in 2 unrelated families.

Five variants c.122A>C (p.Lys41Thr), c.56-30G>A, c.178G>A (p.Glu60Lys), c.229A>C (p.Lys77Gln), and c.206A>T (p.Glu69Val) found in heterozygous individuals were classified as likely benign/benign/VUS. Other variants which were found in homozygous patients were likely pathogenic/pathogenic variants according to ACMG 2015 (Table 3).

### 3.3. Distribution of the Variants

In European subjects, 15 variants were detected, with c.10C>T and c.177C>A each being identified in two unrelated families. Of these variants, 7 were PTC. A total of 7 variants were detected in Asian patients, of which 3 were PTC. Similarly, 3 variants were detected in American patients, all of which were PTC. In the same way, only 1 variant was detected in each of the Oceanic and African populations (Table 2).

### 3.4. Molecular Findings and In Silico Analysis

**Figure 3:**
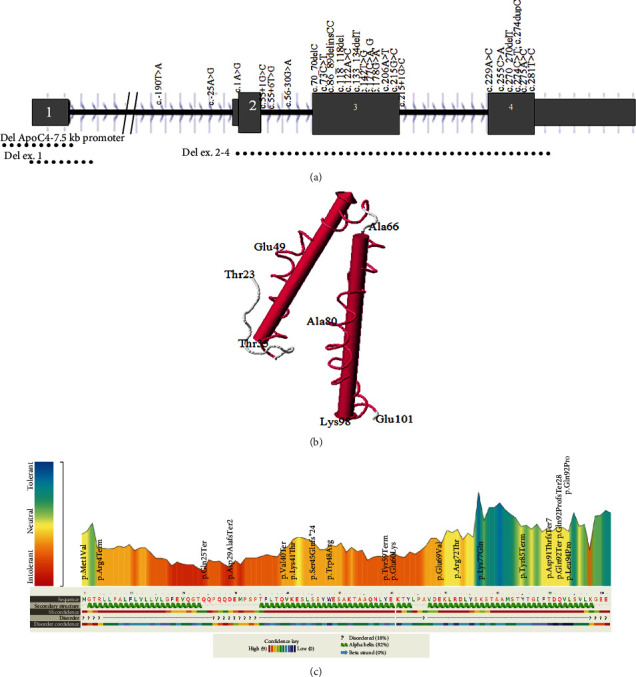
(a) Distribution of pathogenic variants in the predicted secondary structure of the protein. (b) Global structure and dynamics of human apolipoprotein CII using c1o8tA_ template. (c) Pathogenicity analysis of *APOC2* variants through aggregation of homologous human protein domains using MetaDome and Phyre2.

Pathogenicity analysis of *APOC2* variants through aggregation of homologous human protein domains was done using MetaDome (Figure 3). As shown in Figure 3, 14 variants were located in alpha helices. The majority of variants (12 variants) were in exon 3.

## 4. Discussion

The monogenic chylomicronemia as an autosomal recessive trait is usually due to the loss of functional mutations in *LPL*, *LMF1*, *GPIHBP1*, *APOC2*, and *APOA5* genes. *APOC2* encodes apolipoprotein C-II which acts as a cofactor for LPL activity. The plasma levels of TG, VLDL, and chylomicron are increased as a hallmark of apolipoprotein C-II deficiency; decreased levels of LDL, IDL, HDL, apoB, and apoA-I concentrations are also observed [15, 16]. The *APOC2* variant is known as the second most common cause of monogenic chylomicronemia [17]. Recent research suggests that *APOC2* variants are responsible for causing hypertriglyceridemia in 3.7% of the Turkish patient population [18]. As mentioned, apoC2 has essential role and acts as a cofactor for LPL so that any defect in this protein leads to accumulation of chylomicrons. Here, genetic variants and phenotypes of 38 homozygous and 39 heterozygous cases are presented to establish the major clinical features and TG and TC levels among patients.

### 4.1. Clinical Features of *APOC2* Homozygotes and Heterozygotes

The most common features of patients with two variants in *APOC2* gene are as follows: pancreatitis (observed in 55% of these patients), lipemia retinalis (28.95%), abdominal pain (23.68%), hepatosplenomegaly (23.68%), and xanthomas (18.42%). These findings imply that when an *APOC2* variant is present, it is likely to correlate with clinical manifestations in accordance with our observations.

Since pathogenic variants in the *APOC2* gene are usually inherited in a recessive fashion, it is anticipated that heterozygous individuals will not exhibit any clinical symptoms. However, it was discovered in our study that approximately 25% of the heterozygous individuals were symptomatic. We have previously reported symptomatic patients with congenital adrenal hyperplasia due to heterozygous pathogenic variants in *CYP21A2* gene [19, 20]. Possible reasons for this observation are as follows: (1) for the variants in regulatory regions, some individuals may harbor variants in distant regions that regulate gene expression, which have not been previously identified. This may occur even in a heterozygous state. (2) In large deletions and duplications, these variants could be undetectable due to technical limitations in some studies. (3) Digenic and triallelic inheritance is possible, whereby variants in other genes may collaborate with the identified variant in causing the disease. This is also known as digenic or triallelic inheritance. (4) As for gain-of-function variants, some variants may act in a dominant manner; however, functional studies are required to confirm this. (5) The possibility exists that environmental factors, in particular related to hyperlipidemia, may further exacerbate symptoms caused by genetic variants in heterozygous individuals, leading to enhanced lipid levels. On the contrary, it is possible that asymptomatic homozygotes, which comprise approximately 18% of biallelic cases, are the result of environmental and genetic modifying factors as well; these factors may contribute in compensating for *APOC2* deficiency, leading to no clinical symptoms despite increased levels of TG. Takase et al. revealed a Japanese patient who was diagnosed with significantly reduced plasma apoC-II levels, yet they did not find a causative variant present in the *APOC2* gene [21]. This suggests that unidentified factors, such as alternative genes or agents, might play a role in the development of *APOC2*-hypertriglyceridemia.

Pancreatitis has been previously reported in homozygous case reports with both missense and PTC variants [18, 22, 23], but to our knowledge, there has not been a study that defines how common pancreatitis is among homozygous patients. In our study, we found that more than half of the cases showed evidence of this condition. Different types of variants may lead to various forms of HTG with different severity in clinical features. PCT variants usually result in more severe HTG and clinical phenotypes; the mean age of patients with such variants is less than that of patients having missense ones (Table 4). PTC variants in *APOC2* are more common rather than missense variants. Human apoC2 protein has three amphipathic helices, with the C-terminal helix responsible for LPL activation. The majority of the variants is distributed on alpha helices of the protein indicating the important roles of these regions. The exact location of binding site on LPL for the activating cofactor, apolipoprotein C2, and its associated mechanisms have been ambiguous. Kumari et al. demonstrated that APOC2's C-terminal alpha helix interacts with regions of LPL near the catalytic pocket [24]. The findings by Kumari et al. indicate that these alpha helices have an important role in APOC2 and variations in this region potentially cause pathogenic effects, leading to dyslipidemia. To develop effective therapeutic modalities and design cell- and molecular-based therapies, attention to the roles and the structures of different domains of this protein could be helpful.

The *APOC2* variants have been reported from many populations worldwide, although we could not see any variant in Iranian patients which is similar to Oman population [25]. *APOC2* variants may be extremely rare in these populations although it is recommended to study more patients to find its frequencies in these countries. The frequency of *AOPC2* variants is apparently more than other continents although it could be due to that more people have been studied from the continent. The type and frequencies of the variants can vary greatly among different cohorts. Moreover, significant genetic differences may result in different clinical presentation, especially regarding lipid profiles. In previous research, we have reported the impact of both common and population-specific variants on the incidence of other disorders [26–28], although the frequency and effects of different variants in *APOC2* gene have not been published yet. Various *APOC2* variants have been reported across different populations, which imply that certain positions in the locus may be considered a hotspot for alteration. For instance, c.274C>T has been found among patients in both Europe and the United States. Conversely, some variants have been documented solely within specific demographics, denoting the possibility of a founder effect; for example, c.177C>A was detected among two unrelated Italian patients.

### 4.2. Genetic Variant Distribution along the Protein

The human pro-apoliporpotein C-II protein contains 101 amino acids in which amino acids 1-22 are signal peptide. Apolipoprotein C-II has 3 amphipathic alpha helices spanning the approximate residues 16-38, 45-57, and 65-74 (Table 5) [29]. The majority of the variants were in these regions (Table 5 and Figure 3). The lipid-binding and LPL-binding domains of the protein are localized to a region in the N-terminal and C-terminal region, respectively [30, 31]. It has been known that some residues (63, 66, 69, and 70) are important for LPL activation [32].

Since we did not directly participate in patient selections, clinical evaluations, or technical experiments, there were limitations that we should keep in mind, such as the possibility of having missed patients with *APOC2* variants, even during our search process. Future research involving a wider pool of patients or even a multicenter study is needed to provide a more comprehensive examination of the APOC2 variants, eliminating the limitations we encountered during our study. Nevertheless, our study is the first of its scale to study the connection between *APOC2* variants and their respective phenotypes.

## 5. Conclusion

The genetics of *APOC2*-related HTG is more complex. Up to now, thirty phenotype-causing variants in this gene have been reported. Pathogenic variants of this gene cause very severe HTG. PCT variants are expected to consequently lead to more severe phenotypes, while missense variants cause milder phenotypes. *APOC2* variants are distributed in many populations worldwide. Given the fact that most pathogenic variants of this gene reside in alpha helices, these domains could potentially be used for therapeutic purposes in future research endeavors. We have emphasized the genetic architecture associated with APOC2-related hypertriglyceridemia, which can potentially pave the way for more effective preventive strategies and therapeutic interventions.

## Figures and Tables

**Table 1:** TG and TC levels of heterozygous individuals.

No. of family	No. of patient	Nucleotide change	TG	TC	Sex	Population	Ref
1	1	c.122A>C	645	290	M	Anglo-Saxon	[33]
	2	c.122A>C	328	205	M	Anglo-Saxon	[33]
2	3	c.122A>C	750	195	M	Polish	[33]
3	4	c.122A>C	1116	250	M	Anglo-Saxon	[33]
4	5	c.122A>C	135	274	M	Dutch	[33]
5	6	c.122A>C	166	244	M	US	[34]
	7	c.122A>C	74	270	F	US	[34]
	8	c.122A>C	74	151	M	US	[34]
6	9	c.178G>A	282	313	M	Caucasian	[35]
7	10	c.178G>A	203	345	F	African-American	[35]
8	11	c.178G>A	1000	345	M	American	[35]
9	12	c.56-30G>A	1009.7	NA	NA	European	[36]
10	13	c.274dupC	NA	NA	F	European	[1]
11	14	c.206A>T	NA	NA	F	US	[37]
12	15	c.229A>C	780	353	F	African	[38]
	16	c.229A>C	1320	530	F	African	[38]
13	17	c.56-4G>C	5000	298	M	Canada	
14	18	c.177C>A	54	199	M	Italy	[39]
	19	c.177C>A	120	122	F	Italy	[39]
	20	c.177C>A	101	210	M	Italy	[39]
	21	c.177C>A	91	260	F	Italy	[39]
15	22	c.281T>C	248	174	M	Pakistani	[40]
	23	c.281T>C	593.4	58.39	F	Pakistani	[40]
16	24	c.-86A>G	67	163	M	Greek	[41]
	25	c.-86A>G	151	195	F	Greek	[41]
	26	c.-86A>G	150	148	M	Greek	[41]
	27	c.-86A>G	229	202	M	Greek	[41]
17	28	c.86delA+insCC	115.15	195.3	M	Chinese	[22]
	29	c.86delA+insCC	73.52	159.32	F	Chinese	[22]
	30	c.86delA+insCC	69.09	158.93	F	Chinese	[22]
	31	c.86delA+insCC	45.17	143.85	M	Chinese	[22]
	32	c.86delA+insCC	104.52	181.36	M	Chinese	[22]
18	33	2978 bp del	56.69	150.8	F	Bosniak	[42]
	34	2978 bp del	243.6	251.35	M	Bosniak	[42]
19	35	c.215+1G>C	150	250	M	Japanese	[42]
	36	c.215+1G>C	99	181	F	Japanese	[42]
20	37	7.5 kb del	92.12	213.84	M	Netherlands	[43]
	38	7.5 kb del	157.66	279.97	F	Netherlands	[43]
	39	7.5 kb del	171.83	242.46	M	Netherlands	[43]

7.5 kb del: APOC4-promoter+ex. 1; 2978 bp del: ex. 2-4; NA: not available; F: female; M: male.

**Table 2:** The characteristics of patients with defined homozygous variants in the *APOC2* gene.

No. of family	Consanguinity	No.	Nucleotide change	Amino acid	AS	HSM	LR	EX	Pan	AP	TG	TC	Sex	Age (yrs)	Population	Ref
1	C	1	c.10C>T	p.Arg4Ter	+						1796	153	F	0.33	France	[44]
2	NC	3	c.10C>T	p.Arg4Ter			+				8000	770	F	0.12	Hispanic infant	[45]
3	NC	2	c.1A>G	p.Met1Val	+						950	150	F	27	Black-France	[46]
4	C	4	c.142T>G	p.Trp48Arg					+	+	910	140	M	36	Japan	[47]
5	NC	5	c.177C>A	p.Tyr59Ter		+	+	+		+	2020	235	M	41	Italy	[39]
		6	c.177C>A	p.Tyr59Ter	+						1985	183	F	39	Italy	[39]
6	NA	7	c.177C>G	p.Tyr59Ter				—	—	+	2715		F	8	Italy	[48]
		8	c.177C>G	p.Tyr59Ter							2714		F	2	Italy	[48]
7	C	9	c.255C>A	p.Tyr85Ter		+	+				28850	1200	F	0.12	New Zealand Maori	[49]
		10	c.255C>A	p.Tyr85Ter		+	—				16800	850	F	0.06	New Zealand Maori	[49]
8	NA	11	c.274C>T	p.Gln92Ter							NA	NA	NA	—	Venezuelan	[50]
9	NA	12	c.274C>T	p.Gln92Ter			—	+	—	—	1488	626.5	M	48	European	[51]
10	C	13	c.215G>C	p.Arg72Thr					+		1711	455	M	30	Sudan	[23]
11	C	14	c.281T>C	p.Leu94Pro			—	+	—	—	7369	502.7	F	0.12	Pakistani	[40]
		15	c.281T>C	p.Leu94Pro	+		—	—	—	—	2524.4	232	F	3	Pakistani	[40]
12	C	16	c.55+1G>C	Splicing			NA	NA	NA	NA	1848	181	F	30	Turkish	[52]
13	C	17	c.-86A>G	Regulatory					+		1670	198	F	42	Greek	[41]
		18	c.-86A>G	Regulatory					—	—	2995	204	M	16	Greek	[41]
14	C	19	c.70_70delC	p.Gln24AsnfsTer17	+		—	—	—	—	1094	161	M	13	Japanese	[50]
		20	c.70_70delC	p.Gln24AsnfsTer17	+		—	—	—	—	1090	204	F	15	Japanese	[50]
15	NC	21	c.118_118delG	p.Val40Ter		+	+	—	—	+	455.3	100.54	F	22	Netherlands	[53]
		22	c.118_118delG	p.Val40Ter		+	+	+	+	+	2896.4	487.2	M	24	Netherlands	[53]
		23	c.118_118delG	p.Val40Ter		+	+	—	+	—	3360.5	549.1	M	21	Netherlands	[53]
16	C	24	c.270_270delT	p.Asp91ThrfsTer7				—	+	+	3660	500	M	44	Caribbean Islands	[54]
17	NC	25	c.86delA+insCC	p.Asp29AlafsTer2		+	—	+	+		1993	144.62	F	19	Chinese	[22]
18	C	26	2978 bp del (ex. 2-4)	Gross deletion				—	—		4659	529.8	F	0.29	Bosniak	[42]
19	NA	27	7.5 kb del	Gross deletion				—	+		3764.4	358.5	F	31	The Netherlands	[43]
		28	7.5 kb del	Gross deletion	+						1116	120.26	F	27	The Netherlands	[43]
20	C	29	c.73C>T	p.Gln25Ter		+	+	—	—		6295	NA	F	0.12	Turkish	[55]
21	C	30	c.55+6T>G	Splicing		+	+	—	—		4520	NA	F	0.83	Turkish	[55]
22	C	31	c.133_134delTC	p.Ser45Glnfs∗24			—	—	+	+	1148	153	F	50	Colombian	[56]
23	NC	32	c.215+1G>C	Splicing			+	+			12020		M	7	Japanese	[42]
24	NA	33	c.-190T>A	Regulatory				+			4429		M	0.08	Chinese	[57]
25	NA	34	Exon 1 duplication	Gross duplication									NA	—	Japan	[58]
		35	Exon 1 duplication	Gross duplication									NA	—	Japan	[58]
26	NA	36	Exon 1 deletion	Gross deletion					+		1254	188	F	56	Canada	[59]
27	C	37	c.274insC	p.Gln92Pro			+	—	+	+	1325	190	F	58	Anglo-Saxon	[60]
		38	c.274insC	p.Gln92Pro			+	—	+	+	788	87	M	62	Anglo-Saxon	[60]

HSM: hepatosplenomegaly; AS: asymptomatic; LR: lipemia retinalis; EX: xanthomas: Pan: pancreatitis; AP: abdominal pain; NA: not available; F: female; M: male; 7.5 kb del: APOC4-promoter+ex.1.

**Table 3:** General mutations present in the apoc2 gene reported and their bioinformatic evaluation.

No.	Nucleotide change	AA change	Homozygous patients (no.)	Mutation type	MutationTaster	PolyPhen-2 (score)	VarSome	CADD score
1	c.1A>G	p.Met1Val	2	Missense	DC	PD (0.956)	P	20.9
2	c.122A>C	p.Lys41Thr	het	Missense	DC	B (0.033)	LB	17.33
3	c.142T>G	p.Trp48Arg	1	Missense	DC	PD (1.000)	LP	24.6
4	c.178G>A	p.Glu60Lys	het	Missense	DC	B (0.005)	LB	2.477
5	c.215G>C	p.Arg72Thr	1	Missense	DC	PD (0.992)	P	34
6	c.281T>C	p.Leu94Pro	1	Missense	Polymorphism	PD (0.986)	VUS	20.1
7	c.206A>T	p.Glu69Val	het	Missense	Polymorphism	PD (0.952)	VUS	23.6
8	c.229A>C	p.Lys77Gln	het	Missense	Benign	PD (0.664)	B	NA
9	c.10C>T	p.Arg4Ter	1	Nonsense	DC	NA	P	33
10	c.177C>A	p.Tyr59Ter	1	Nonsense	DC	NA	P	33
11	c.177C>G	p.Tyr59Ter	1	Nonsense	DC	NA	P	34
12	c.255C>A	p.Tyr85Ter	1	Nonsense	DC	NA	P	34
13	c.274C>T	p.Gln92Ter	2	Nonsense	DC	NA	P	38
14	c.73C>T	p.Gln25Ter	1	Nonsense	DC	NA	LP	34
15	c.70_70delC	p.Gln24AsnfsTer17	1	Small deletions	DC	NA	LP	NA
16	c.118_118delG	p.Val40Ter	1	Small deletions	DC	NA	P	NA
17	c.270_270delT	p.Asp91ThrfsTer7	1	Small deletions	DC	NA	LP	NA
18	c.274dupC	p.Gln92ProfsTer28	1	Small insertions	DC	NA	P	NA
19	c.86delA+insCC	p.Asp29AlafsTer2	1	Small indels	DC	NA	LP	NA
20	c.133_134delTC	p.Ser45Glnfs∗24	1	Small deletions	DC	NA	P	NA
21	c.55+1G>C	—	1	Splicing	DC	NA	P	32
22	c.56-30G>A	—	het	Splicing	Polymorphism	NA	B	2.504
23	c.55+6T>G	—	1	Splicing	DC	NA	LP	22.5
24	c.215+1G>C	—	1	Splicing	DC	NA	P	31
25	c.-190T>A	—	1	Regulatory	NA	NA	NA	NA
26	c.-86A>G		1	Regulatory	NA	NA	NA	NA
27	2978 bp del (ex. 2-4)	—	1	Gross deletions	NA	NA	NA	NA
28	7.5 kb del	—	1	Gross deletions	NA	NA	NA	NA
29	Duplication of exon 1	—	1	Gross dup.	NA	NA	NA	NA
30	Exon 1 deletion	—	1	Gross deletions	NA	NA	NA	NA

B: benign; LB: likely benign; PD: probably damaging; NA: not available; P: pathogenic; LP: likely pathogenic; VUS: variant of uncertain significance; 7.5 kb del*: APOC4*-promoter+ex. 1.

**Table 4:** The main clinical feature of patients based on their variants and distributions in different continents.

Variant type	Patients	Families	HSM	AS	LR	EX	Pan	AP	TG	TC	Sex ratio M (F)	Age (yrs)	European	America	Asia	Oceania	Africa
Termination (del, nonsense)	23	16	8	6	7	4	7	6	4506.98	395.45	6 (16)	20.96	15	3	3 (eastern)	2	0
Missense	7	5	0	2	2	1	4	3	2225.34	250.96	3 (4)	30.87	3	0	3	0	1
Spl+Reg	6	5	1	2	2	2	1	0	4580.33	194.33	3 (3)	15.98	4	0	2	0	0
Gross duplication	2	1	NA	NA	NA	NA	NA	NA	NA	NA	NA	NA	0	0	2	0	0

HSM: hepatosplenomegaly; AS: asymptomatic; LR: lipemia retinalis; EX: xanthomas: Pan: pancreatitis; AP: abdominal pain.

**Table 5:** Molecular features of the protein.

Region	Position(s) of amino acid	No. of variants				
		Missense	PCT	Gross del/dup	Intronic	Regulatory
Regulatory region	—	—	—	—	—	2
Signal peptide	1-22	1	1	3	3	—
O-Glycosylated at one site (alpha helix)	23-38 (16-38)	0	3		0	—
Chain (alpha helix)	39-65 (45-57)	3	4		0	—
Lipid binding (alpha helix)	66-74 (65-74)	2	0	1	1	—
Chain	75-77	1	0		0	—
Lipoprotein lipase cofactor	78-101	1	4		0	0
Total		8	12	4	4	2

## Data Availability

All data generated or analyzed during this study are included in this published article.
